# Styles of Delivering News About a Child’s Cancer and Parents’ PTSD Symptoms

**DOI:** 10.1192/j.eurpsy.2026.10938

**Published:** 2026-07-31

**Authors:** M. Farchi

**Affiliations:** Social Work, Tel-Hai University on the rise, Upper Galilee, Israel

## Abstract

**Introduction:**

Receiving a child’s cancer diagnosis is a highly traumatic experience for parents, often leading to significant psychological distress, including symptoms of Post-Traumatic Stress Disorder (PTSD). The way healthcare professionals deliver this news can affect the severity of parents’ reactions. While some research examines communication style’s impact on patients, few studies focus on its effects on parents. This study examines how oncologists’ communication styles affect parents’ psychological outcomes after a child’s cancer diagnosis.

**Objectives:**

This study explores the relationship between the communication style used by oncologists when delivering a child’s cancer diagnosis and the subsequent levels of PTSD symptoms, mental resilience, and self-efficacy in parents.

**Methods:**

Communicating Questionnaire (SCQ), PTSD Checklist for DSM-V (PCL-5), the Connor-Davidson Resilience Scale (CDRISK), and the General Self-Efficacy Scale. Correlations and hierarchical multiple regressions were performed to examine the relationship between communication style and psychological outcomes

**Results:**

Parents who perceived the oncologist’s communication style as more activating (clear, structured, and action-oriented) reported significantly lower levels of PTSD symptoms and higher levels of resilience and self-efficacy. The perception of empathy played a crucial role, particularly when physicians balanced emotional and cognitive empathy. This balance was linked to better psychological outcomes in parents

**Conclusions:**

The study highlights the critical role of communication style in mitigating the psychological impact of a child’s cancer diagnosis on parents. Training healthcare providers to balance cognitive and emotional empathy in communication may reduce PTSD symptoms and enhance resilience and self-efficacy in parents, ultimately improving their psychological well-being during such a challenging time.

**Disclosure of Interest:**

None Declared

